# Tumor‐infiltrating CD8^+^ T‐cell density is an independent prognostic marker for oral squamous cell carcinoma

**DOI:** 10.1002/cam4.1889

**Published:** 2019-01-01

**Authors:** Shota Shimizu, Hiroyoshi Hiratsuka, Kazushige Koike, Kei Tsuchihashi, Tomoko Sonoda, Kazuhiro Ogi, Akira Miyakawa, Junichi Kobayashi, Takeshi Kaneko, Tomohiro Igarashi, Tadashi Hasegawa, Akihiro Miyazaki

**Affiliations:** ^1^ Department of Oral Surgery Sapporo Medical University School of Medicine Sapporo Japan; ^2^ Department of Public Health Sapporo Medical University School of Medicine Sapporo Japan; ^3^ Department of Surgical Pathology Sapporo Medical University School of Medicine Sapporo Japan

**Keywords:** oral squamous cell carcinoma, prognostic marker, survival, tissue compartment, tumor‐infiltrating CD8^+ ^T cells

## Abstract

**Background:**

The presence of tumor‐infiltrating lymphocytes (TILs) is associated with improved survival in head and neck squamous cell carcinoma. However, the prognostic value of TILs remains unclear in oral squamous cell carcinoma (OSCC).

**Methods:**

We evaluated the associations between tumor‐infiltrating CD8^+^ T‐cell density and survival in five distinct compartments in 139 OSCC cases.

**Results:**

There was a significant association between increased tumor‐infiltrating CD8^+^ T cells and their distribution. High parenchymal CD8^+^ T‐cell density at the invading tumor edge was associated with improved overall survival (OS) and disease‐specific survival (DSS; *P* < 0.01 and *P* < 0.01, respectively). High stromal CD8^+^ T‐cell density at the tumor periphery was also associated with improved recurrence‐free survival (RFS; *P* < 0.01). Cox regression analysis revealed that high stromal CD8^+^ T‐cell density at the tumor periphery and high parenchymal CD8^+^ T‐cell density at the invading edge were independent prognostic makers (hazard ratio: 0.38 and 0.19, 95% confidence interval, 0.18‐0.80 and 0.05‐0.72, *P* = 0.01 and 0.01, respectively) for RFS and OS, respectively.

**Conclusions:**

Assessment of CD8^+^ T cells at the parenchyma of the invading edge and peripheral stroma provides an indicator of tumor recurrence and prognosis.

## 1 INTRODUCTION

The oral cavity is a distinct site in the head and neck region, and oral cavity cancers account for 3% of all cancers. Oral cancer is a minor subset of head and neck cancer and is drastically different in etiology, management and prognosis from other head and neck cancers. For instance, oral squamous cell carcinoma (OSCC) is associated with distinct clinical characteristics showing unfavorable prognosis compared to human papilloma virus‐positive oropharyngeal cancer.^1^

Tumor‐infiltrating lymphocytes (TILs) have been described as a prognostic factor in various types of cancers.^2^, ^3^, ^4^ In head and neck squamous cell carcinoma (HNSCC), the presence of TILs indicates a favorable prognosis,^5^ and TILs at periphery of the tumor is a positive prognostic marker for patients with OSCC.^6^

Functional analysis of TILs in rats showed that T‐cells infiltrating in the tumor tissues have effects on tumor rejection,^7^ suggesting the presence of host antitumor immunological resistance and a key role for cell‐mediated immunity in tumor immunology. Additionally, the immune infiltrate of human tumors is mainly composed of lymphocytes, with T cells being the predominant cell type.^8^, ^9^, ^10^ Therefore, the immune system plays a key role in the control of tumor growth and progression.^11^ Moreover, an abundance of T‐cell infiltrates is associated with favorable clinical outcome in many types of cancers, including head and neck cancer,^12^ and the exclusion of CD8^+^ T cells from the vicinity of cancer cells in colorectal tumors correlates with a poor long‐term clinical outcome.^13^ Patients with HNSCC with high tumor infiltration of CD8^+^ T cells have significantly better outcomes compared to those with lower or no infiltration.^14^ Few studies, however, have examined the predictive impact of CD8^+^ T‐cell infiltration in OSCC. It has been reported that a high TIL density is significantly associated with favorable prognosis, although the prognostic value of tumor‐infiltrating CD8^+^ T cells remains controversial.^15^, ^16^ The location of predictive immune cell infiltrates in various tissue compartments remains unclear, and tumor‐infiltrating immune cell infiltrate is not homogeneous in OSCC. Oguejiofor et al^17^ showed on multivariate analysis that a high density of CD8^+^ T cells in the tumor stroma, but not the tumor parenchyma, was associated with significantly better overall survival (OS) in human papilloma virus‐positive oropharyngeal SCC, whereas Nguyen et al^18^ observed that the tumor parenchymal, at not tumor invasive front, CD4^+^ and CD8^+^ lymphocytes influence their prognostic impact. On the other hand, Balermpas et al^19^ emphasized that although high stromal density of CD8^+^ T cells was a positive prognostic factor for local failure‐free survival (LFFS), distant metastases‐free survival (DMFS), progression‐free survival (PFS), OS, high density of parenchymal CD8^+^ T cells correlated only with better DMFS and OS, and high density of peripheral CD8^+^ T cells correlated only with better PFS and LFFS. Thus, past studies could not clearly demonstrate the predictive impact of CD8^+^ T‐cells infiltration. Therefore, it is necessary to examine heterogeneous compartments within the tumor microenvironment. The authors have emphasized in a previous paper that T cells in OSCC samples preferentially accumulate at tumor stroma and the tumor periphery.^9^ Because the invasive tumor edge is the first line of defense against cancer proliferation and metastasis, these observations have led to the hypothesis that direct and indirect effector CD8^+ ^T cells infiltrate the parenchyma or stroma of different anatomical areas, such as the center of the tumor, invading tumor edge, or tumor periphery.

The aim of this study was to conduct immunohistochemical evaluation of the density and location of five types of CD8^+^ cytotoxic T‐cell infiltrates, including those located in stroma in the center of the tumor, parenchyma in the center of the tumor, stroma in the invading tumor edge, parenchyma in the invading tumor edge, and periphery of the tumor, to investigate their prognostic relevance in OSCC.

## 2 PATIENTS AND METHODS

### 2.1 Patients and tissue samples

Diagnostic tissue blocks from previously untreated patients who were diagnosed with OSCC and underwent definitive surgery between January 2004 and December 2014 at the Sapporo Medical University Hospital were used in this study to investigate the pretherapeutic immune response in OSCC. None of the patients received any form of neoadjuvant therapy prior to surgery, and no patients received adjuvant therapy, excluding palliative chemo‐ and/or radiotherapy. Patients with distant metastases at initial physical examination were also excluded. All tissue specimens were embedded in paraffin and processed routinely.

### 2.2 Immunohistochemical and histological staining

Immunohistochemistry was employed to detect CD8^+^ T cells in the surgical specimens. Briefly, 4‐µm serial sections from paraffin‐embedded samples deparaffinized in xylenes were soaked in 10 mmol/L citrate buffer (pH 8.0) and placed in an autoclave at 121°C for 10 minutes for antigen retrieval. Endogenous peroxidase was blocked by incubation with 0.3% hydrogen peroxide in methanol for 30 minutes. The sections were then incubated with primary monoclonal antibody targeting CD8 (Clone C8/144B; Code 413201; Nichirei Bioscience, Inc, Tokyo, Japan) at 4°C overnight. Secondary antibodies were applied as indicated by the EnVision^+^ system (EnVision^+^; Code K5007; HRP; Rabbit/Mouse; DAKO, Glostrup, Denmark) manufacturer instructions. Staining was visualized with diaminobenzidine tetrachloride. The sections were counterstained with hematoxylin, dehydrated, cleared, and mounted. In negative controls, the primary antibody was omitted.

### 2.3 Histopathological and immunohistopathological evaluations

**Figure 1:**
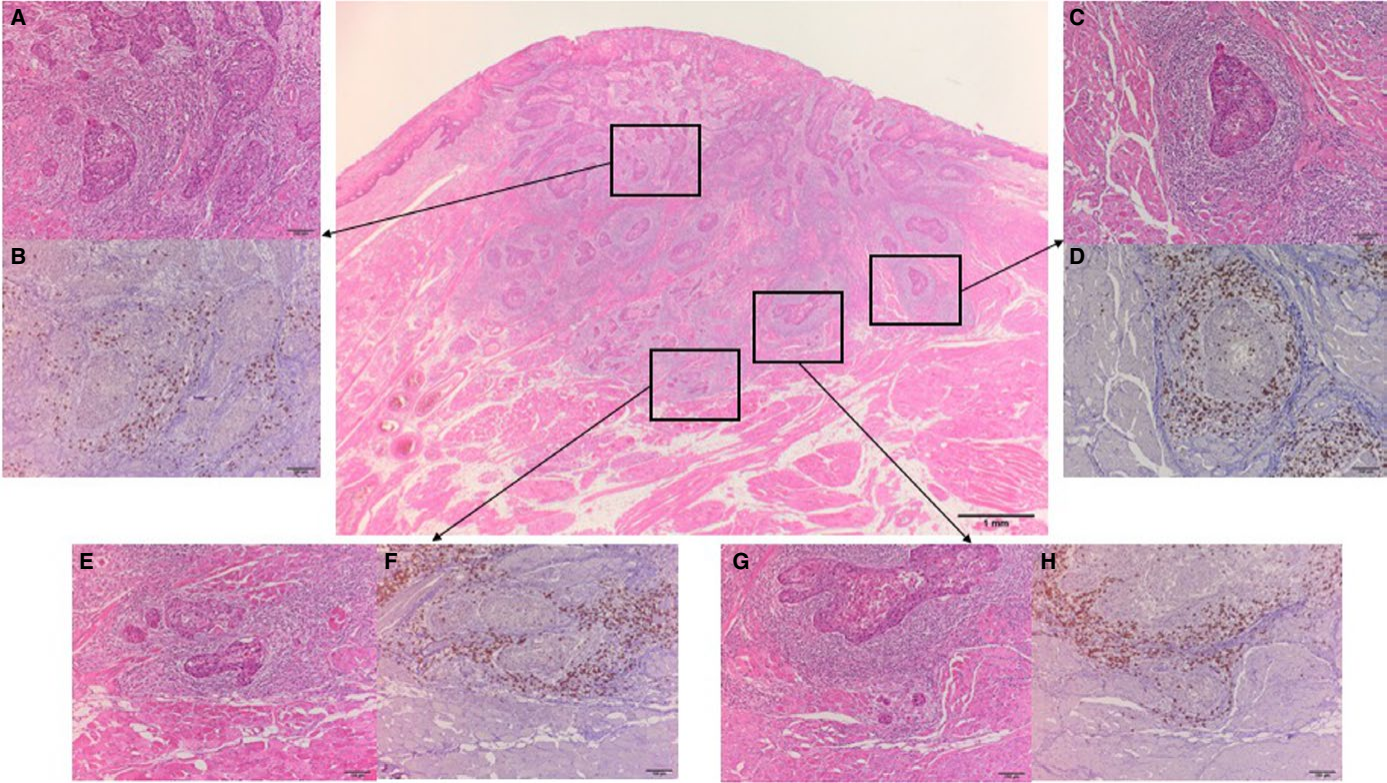
Representative hematoxylin and eosin (H&E) and CD8 immunohistochemical (IHC) staining of oral squamous cell carcinoma sections for assessment of CD8^+^ T‐cell density at five different anatomic locations; the parenchyma and stroma in the center of the tumor (A, H & E; B, IHC), the parenchyma and stroma in the invading tumor edge (C, H & E; D, IHC), and the periphery of the tumor (E, H & E; F, IHC; G, H & E; H, IHC). The invading edge is a belt zone including a tumor nest layer inside the tumor border. The periphery of the tumor is outside of the tumor border. Evaluation of peripheral CD8^+^ T‐cell density included the area of most scattered cancer cells or small islands (G and H rather than E and F). The regions in the rectangle (A, C, E, and G) are shown at ×100 magnification of the arrowed panel

As the ultimate interest of this study was to analyze cytotoxic CD8^+^ T cells together, distinguished based on their five different locations, CD8^+^ T cells were evaluated in the following five different areas of the tumor: the tumor parenchyma (within the cancer cell nests) and tumor stroma at the intratumoral center and invading tumor edge (inside of the tumor‐host interface), and the periphery of the tumor (Figure 1). CD8^+^ T‐cell density was quantitatively assessed. After the CD8^+^ T cells were identified in the five locations at low magnification, they were counted manually in the areas of highest CD8^+^ intensity under 400x magnification, and cell counts were averaged. Tumor areas with crush artifacts, necrosis, or apoptosis were excluded from analysis. The CD8^+^ T cells at the invading tumor edge were estimated from small clusters of forming cancer cells or nests at deepest invading margin. For assessment of tumor‐infiltrating CD8^+^ T‐cell density in each compartment, at least three random fields were viewed, and, in cases of heterogeneity, the calculation that was most representative of the entire section was assigned. The analysis of CD8^+^ T cell density was assessed using DP2‐BSW software for an Olympus Microscope Digital Camera (Olympus Co., Tokyo, Japan) by three of the authors (SS, KK, and AM) simultaneously. The mean T‐cell density for each compartment was used to stratify patients into high and low CD8^+^ T‐cell density groups. The prognostic role of CD8^+^ T‐cell density was analyzed for each of the five different tumor areas. During the assessment of tumor‐infiltrating CD8^+^ T‐cell density, the labels bearing the patient's names were covered. The relationships between CD8^+^ T‐cell density and clinicopathological findings were also examined. Histopathologic findings were tabulated from information in routine histopathologic reports. The tumor extent and the histopathological grading were classified according to the 7th version of the American Joint Committee on Cancer TNM staging system.^20^

### 2.4 Statistical analysis

For nonparametric distribution of samples, *P*‐values were calculated by the Wilcoxon‐Mann‐Whitney test. Associations between the density of CD8^+^ T‐cell infiltration and clinicopathological findings were evaluated using Fisher's exact test or chi‐square test. Disease‐specific survival (DSS) was calculated from the date of definitive surgery to death with the tumor. OS was defined from the date of definitive surgery to death from any cause. Recurrence‐free survival (RFS) was defined from the date of definitive surgery to locoregional or distant tumor recurrence or death from any cause. DSS, OS, and RFS were calculated using the Kaplan‐Meier method and compared using the log‐rank test for each group. Two‐tailed *P*‐values <0.05 were considered to indicate statistical significance. Variables that had prognostic potential in univariate analysis were subjected to multivariate analysis with Cox proportional hazard regression models. The Statistical Package for Social Sciences (SPSS), version 23.0, software for Windows (IBM Corp., Armonk, NY, USA) was used for statistical analysis.

## 3 RESULTS

### 3.1 Patient and tumor characteristics

Between January 2004 and December 2014, a total of 221 primary patients were treated by curative surgery. Of these, 139 patients were treated without neoadjuvant therapy and had sufficient tissue sample for further analysis. The mean age of the patients at diagnosis was 67 years (range 33‐93 years, median 69 years), and 55.4% of the patients were males. Sixty‐four percent of the patients had carcinoma of the tongue and floor of the mouth, and 74% of the patients had early‐stage OSCC. The 5‐year DSS, OS, and RFS were well differentiated according to disease stage: DSS in stage I, stage II, and stage III/IV were 100%, 91,5%, 73.5%, respectively (*P* = 0.01); OS was 97.1%, 82.9%, 63.3%, respectively (*P* < 0.01); and RFS was 71.1%, 63.6%, 49.5%, respectively (*P* = 0.12).

### 3.2 Tumor‐infiltrating CD8^+^ T‐cell density

**Figure 2:**
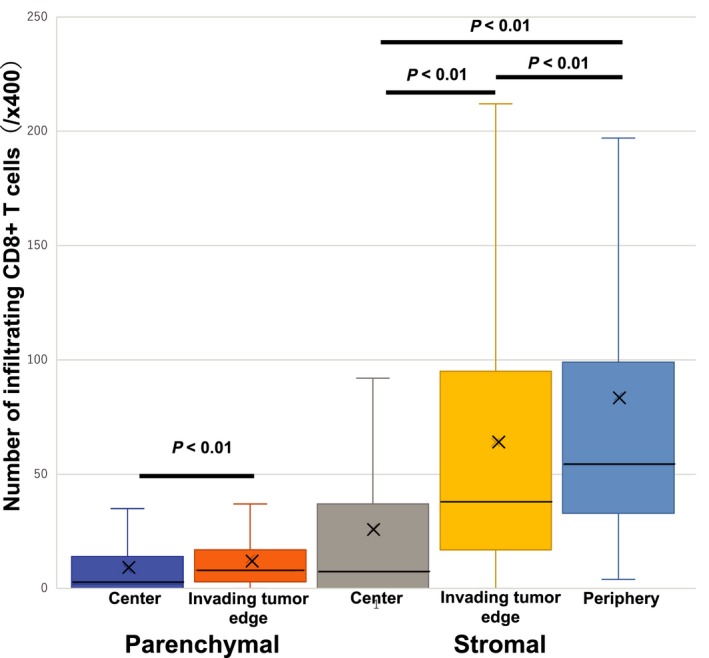
CD8^+^ T‐cells density. The cell density was evaluated in the parenchyma (within the tumor nests) at the center of the tumor and the invading tumor edge, and in the stroma at center of the tumor, invading tumor edge, and periphery of the tumor. The density of the cells indicates the number of positive cells per ×400 microscopic field. Histograms represent the mean plus/minus standard error of cell densities. The *X* represents the average of each histogram. The *Y* represents the number of infiltrating CD8^+^ T cells

**Figure 3:**
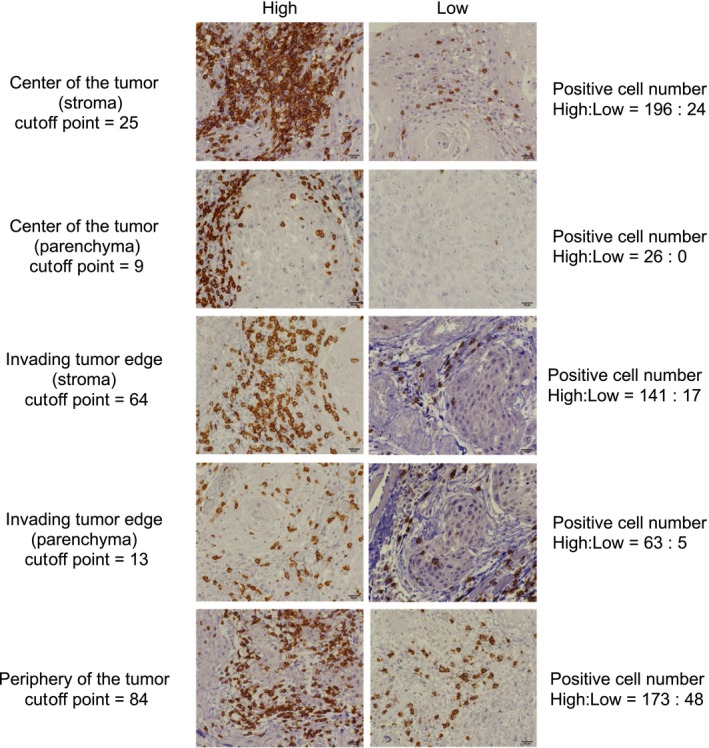
Representative examples of low and high CD8^+^ T‐cell densities in oral squamous cell carcinoma samples. Magnification, ×400

All tumor samples had heterogeneous staining for CD8^+^ T cells in the parenchymal, stromal, and peripheral regions. The distributions of CD8^+^ T cells were extremely different from the stromal compartment (at the periphery of the tumor, invading tumor edge, or center of the tumor) to the parenchymal compartment (at the invading tumor edge, or center of the tumor). Analysis of tumor‐infiltrating CD8^+^ T cells revealed that the majority of patients showed some degree of stromal, parenchymal, and peripheral CD8^+^ T‐cell infiltration. The mean numbers of stromal CD8^+^ T cells in the center of the tumor, parenchymal CD8^+^ T cells in the center of the tumor, stromal CD8^+^ T cells in the invading edge of the tumor, parenchymal CD8^+^ T cells in the invading edge of the tumor, and peripheral CD8^+^ T cells were 25 (range, 0‐200; median 5), 9 (range, 0‐90; median 1), 64 (range, 0‐332; median 36), 13 (range, 0‐74; median 8) and 84 (range, 0‐476; median 54), respectively (Table 1). The density of the tumor‐infiltrating CD8^+^ T cells was significantly associated with location in the tumor (Figure 2), suggesting that the number of CD8^+^ T cells significantly number of CD8^+^ T cells significantly increased in the invading edge and periphery of the tumor as compared to the center of the tumor, in both the stroma and parenchyma (*P* < 0.01 and *P* < 0.01, respectively). Based on the results, the host reaction was investigated by using mean cutoff values for each compartment. Figure 3 illustrates the representative examples of low and high parenchymal and stromal CD8^+^ T‐cell infiltration.

**Table 1:** Patient distribution according to locations and densities of tumor‐infiltrating CD8^+^ T cells and clinicopathological variables

Observed locations and findings	Density of tumor infiltrating CD8^+^ T cells and variables	No. of cases	%
Stroma in the center of the tumor (/×400) (range 0‐200)	Cutoff point = 25 cells		
	High; ≧25	51	36.7
	Low; <25	88	63.3
Parenchyma in the center of the tumor (/×400) (range 0‐200)	Cutoff point = 9 cells		
	High; ≧9	42	30.2
	Low; <9	97	69.8
Stroma in the invading tumor edge (/×400) (range 0‐200)	Cutoff point = 64 cells		
	High; ≧64	46	33.1
	Low; <64	93	66.9
Parenchyma in the invading tumor edge (/×400) (range 0‐200)	Cutoff point = 13 cells		
	High; ≧13	50	36.0
	Low; <13	89	64.0
Periphery of the tumor (/×400) (range 0‐200)	Cutoff point = 84 cells		
	High; ≧84	45	32.4
	Low; <84	94	67.6
Clinical findings	Gender		
	Male	77	55.4
	Female	62	44.6
	Age (years)		
	<67	58	41.7
	≧67	81	58.3
	Tumor site		
	Tongue/Floor of mouth	90	64.7
	Others	49	35.3
	cT stage		
	cT1	46	33.1
	cT2	77	55.4
	cT3/4	16	11.5
	cN stage		
	cN0	108	77.7
	cN1/2	31	22.3
	cTNM stage		
	Stage I	42	30.2
	Stage II	61	43.9
	Stage III/IV	36	25.9
	Operative method		
	Peroral tumor excision	92	66.2
	Primary tumor excision with neck dissection	47	33.8
Pathological findings	Histologic grade		
	Grade 1	73	52.5
	Grade 2	61	43.9
	Grade 3	5	4.6
	pT stage		
	pT1	69	49
	pT2	54	38.9
	pT3/4	16	11.5
	pN stage		
	pN0	112	80.6
	pN1/2	27	19.4
	pTNM stage		
	Stage I	58	41.7
	Stage II	45	32.4
	Stage III/IV	36	25.9
	Lymphovascular invasion		
	Absence	116	83.5
	Presence	23	46.5
	Perineural invasion		
	Absence	126	90.6
	Presence	13	9.4
	Surgical margin status		
	Negative	126	90.6
	Positive	13	9.4

### 3.3 CD8^+^ T‐cell density and survival

**Figure 4:**
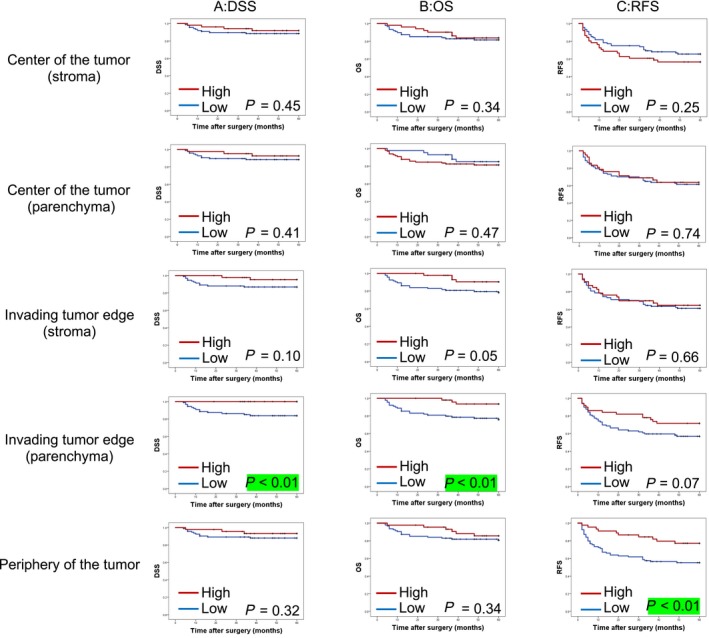
Prognostic role of tumor‐infiltrating CD8^+^ T cells in the outcome of patients with oral squamous cell carcinoma after definitive surgery by density of CD8^+^ T cells. A, Kaplan‐Meier curves for disease‐specific survival (DSS) by location of CD8^+^ T cell density. B, Kaplan‐Meier curves for overall survival (OS) by location of CD8^+^ T‐cell density. (C) Kaplan‐Meier curves for recurrence‐free survival (RFS) by location of CD8^+^ T‐cell density. The red line indicates high CD8^+^ T‐cell density and blue line indicates low CD8^+^ T‐cell density

The median follow‐up period for all patients was 79 months (range 4‐164 months). The primary tumor recurred in 16 patients (11.5%), and regional lymph node relapse was found in 25 patients (18.0%). The 5‐year DSS, OS, and RFS of all patients were 89.6%, 82.4%, and 62.2%, respectively. Patients with OSCC with high CD8^+ ^T‐cell density in the parenchymal invading tumor edge had a significantly superior DSS (100% vs 83.6%, *P* < 0.01) and OS (93.6% vs 76.1%, *P* < 0.01), whereas high CD8^+^ T‐cell density in the parenchyma at the center of the tumor was not associated with improved DSS or OS in univariate analysis (Table 2). Patients with OSCC with high CD8^+^ T‐cell density only in the periphery of the tumor had a significantly superior RFS (77.1% vs 55.1%, *P* < 0.01). CD8^+^ T‐cell density in any other compartment was not associated with RFS, DSS, or OS in univariate analysis (Figure 4). Thus, high stromal CD8^+^ T‐cell infiltration in the periphery of the tumor and parenchyma in the invading tumor edge were significantly associated with DSS, OS, and RFS, whereas high stromal CD8^+^ T‐cell infiltration at other sites was not. Similarly, high parenchymal CD8^+^ T‐cell infiltration in the tumor invading edge also significantly affected DSS and OS, whereas high parenchymal CD8^+^ T‐cell infiltration in the center of the tumor did not (Table 2).

**Table 2:** Five‐year disease‐specific, overall, and relapse‐free survival according to locations and densities of tumor‐infiltrating CD8^+^ T cells and clinicopathological variables

Observed locations and findings	Density of tumor infiltrating CD8^+^ T cells and variables	DSS		OS		RFS	
		Survival rate (%)	Log‐rank test (*P*‐value)	Survival rate (%)	Log‐rank test (*P*‐value)	Survival rate (%)	Log‐rank test (*P*‐value)
Stroma in the center of the tumor (/×400) (range 0‐200)	Cutoff point = 25 cells						
	High; ≧25	92.0	*P* = 0.45	85.5	*P* = 0.34	56.5	*P* = 0.25
	Low; <25	88.3		80.4		65.5	
Parenchyma in the center of the tumor (/×400) (range 0‐200)	Cutoff point = 9 cells						
	High; ≧9	92.6	*P* = 0.41	85.0	*P* = 0.47	63.8	*P* = 0.74
	Low; <9	88.4		81.2		61.5	
Stroma in the invading tumor edge (/×400) (range 0‐200)	Cutoff point = 64 cells						
	High; ≧64	95.5	*P* = 0.10	90.6	*P* = 0.05	64.6	*P* = 0.66
	Low; <64	86.8		78.3		61.1	
Parenchyma in the invading tumor edge (/×400) (range 0‐200)	Cutoff point = 13 cells						
	High; ≧13	100.0	***P* < 0.01**	93.6	***P* < 0.01**	71.6	*P* = 0.07
	Low; <13	83.6		76.1		56.9	
Periphery of the tumor (/×400) (range 0‐200)	Cutoff point = 84 cells						
	High; ≧84	93.2	*P* = 0.32	85.9	*P* = 0.34	77.1	***P* < 0.01**
	Low; <84	87.9		80.7		55.1	
Clinical findings	Gender						
	Male	90.7	*P* = 0.65	82.8	*P* = 0.85	65.9	*P* = 0.26
	Female	88.3		82.0		57.8	
	Age (years)						
	<67	98.2	***P* < 0.01**	94.6	***P* < 0.01**	73.7	***P* = 0.02**
	≧67	83.4		73.7		54.1	
	Tumor site						
	Tongue/Floor of mouth	94.2	***P* = 0.01**	88.7	***P* < 0.01**	66.4	*P* = 0.10
	Others	81.3		70.7		54.5	
	cT stage						
	cT1	97.7	***P* = 0.01**	95.6	***P* < 0.01**	69.0	*P* = 0.10
	cT2	87.9		80.0		62.1	
	cT3/4	74.5		55.6		42.9	
	cN stage						
	cN0	95.2	***P* < 0.01**	87.3	***P* < 0.01**	65.3	*P* = 0.14
	cN1/2	69.6		64.1		51.4	
	cTNM stage						
	Stage I	100.0	***P* = 0.01**	97.1	***P* < 0.01**	71.1	*P* = 0.12
	Stage II	91.5		82.9		63.6	
	Stage III/IV	73.5		63.3		49.5	
	Operative method						
	Peroral tumor excision	96.6	***P* < 0.01**	89.3	***P* < 0.01**	63.3	*P* = 0.61
	Primary tumor excision with neck dissection	76.0		68.6		60.2	
Pathological findings	Histologic grade						
	Grade 1	94.5	*P* = 0.15	87.0	*P* = 0.26	70.3	***P* < 0.01**
	Grade 2	84.5		77.0		55.1	
	Grade 3	80.0		80.0		20.0	
	pT stage						
	pT1	98.5	***P* < 0.01**	93.7	***P* < 0.01**	65.8	***P* = 0.01**
	pT2	88.3		78.4		66.1	
	pT3/4	56.3		47.1		35.3	
	pN stage						
	pN0	96.2	***P* < 0.01**	86.9	***P* < 0.01**	73.8	***P* < 0.01**
	pN1/2	62.7		62.7		14.8	
	pTNM stage						
	Stage I	98.2	***P* < 0.01**	92.7	***P* < 0.01**	74.0	***P* < 0.01**
	Stage II	95.2		83.4		77.1	
	Stage III/IV	68.6		63.7		24.7	
	Lymphovascular invasion						
	Absence	91.2	*P* = 0.22	85.2	*P* = 0.09	66.1	***P* = 0.03**
	Presence	82.4		69.3		43.0	
	Perineural invasion						
	Absence	91.9	***P* < 0.01**	85.4	***P* < 0.01**	65.5	***P* < 0.01**
	Presence	66.1		53.8		30.8	
	Surgical margin status						
	Negative	89.5	*P* = 0.73	84.0	*P* = 0.24	64.1	*P* = 0.38
	Positive	90.9		67.1		42.7	

Bold indicates *P* < 0.05.

The relationships between survival and traditional prognostic factors were also examined. As expected, age (*P* < 0.01), tumor site (*P* = 0.01), cT stage (*P* = 0.01), cN stage (*P* < 0.01), cTNM stage (*P* = 0.01), operative method (*P* < 0.01), pT stage (*P* < 0.01), pN stage (*P* < 0.01), pTNM stage (*P* < 0.01), and presence of perineural invasion (*P* < 0.01) were associated with DSS in univariate analyses. Age (*P* < 0.01), tumor site (*P* < 0.01), cT stage (*P* < 0.01), cN stage (*P* < 0.01), cTNM stage (*P* < 0.01), operative method (*P* < 0.01), pT stage (*P* < 0.01), pN stage (*P* < 0.01), pTNM stage (*P* < 0.01), and presence of perineural invasion (*P* < 0.01) were also associated with OS. Similarly, age (*P* = 0.02), histological tumor grade (*P* < 0.01), pT stage (*P* = 0.01), pN stage (*P* < 0.01), pTNM stage (*P* < 0.01), presence of lymphovascular invasion (*P* = 0.03), and presence of perineural invasion (*P* < 0.01) were also associated with RFS. Thus, clinical parameters, including age, cT stage, cN stage, cTNM stage, and operative method, were significantly associated with OS and DSS. OSCC of tongue and floor of the mouth were also significantly associated with tumor‐related and tumor‐unrelated death (Table 2).

Multivariate analysis was performed, including the CD8^+^ marker at the tumor areas that were significantly associated with survival and clinicopathological parameters that were significant in univariate analysis. A final stepwise model for Cox multivariate analysis supported the advantage of the CD8^+^ T‐cell density as well as clinicopathological features in predicting relapse and survival. As shown in Table 3, parenchymal CD8^+^ T‐cell density at the invading tumor edge (hazard ratio [HR] 0.19, 95% confidence interval [CI] 0.05‐0.72, *P* = 0.01), age (HR 0.26, 95% CI 0.07‐0.98, *P* = 0.04), cN stage (HR 0.17, 95% CI 0.03‐0.95, *P* = 0.04), pT stage (HR 6.6, 95% CI 1.49‐29.96, *P* = 0.01) and pN stage (HR 6.47, 95% CI 1.40‐29.86, *P* = 0.01) were independent prognostic factors for OS. Peritumoral CD8^+^ T‐cell density (HR 0.38, 95% CI 0.18‐0.80, *P* = 0.01), age (HR 0.47, 95% CI 0.24‐0.90, *P* = 0.02) and pN stage (HR 6.09, 95% CI 2.36‐15.71, *P* < 0.01) were independent prognostic factors for RFS.

**Table 3:** Predictive factors associated with DSS, OS, and RFS in univariate and multivariate analyses

Immunohistochemical, clinical, and pathological findings		DSS						OS						RFS					
		Univariate			Multivariate			Univariate			Multivariate			Univariate			Multivariate		
		HR	95%CI	*P*‐value	HR	95%CI	*P*‐value	HR	95%CI	*P*‐value	HR	95%CI	*P*‐value	HR	95%CI	*P*‐value	HR	95%CI	*P*‐value
Immunohistochemical findings	Stroma in the center of the tumor (/×400) (range 0‐200)	0.64	0.20‐2.06	0.46				0.67	0.27‐1.61	0.37				1.48	0.81‐2.46	0.21			
	Parenchyma in the center of the tumor (/×400) (range 0‐200)	0.59	0.16‐2.12	0.42				0.72	0.28‐1.82	0.49				0.93	0.51‐1.70	0.82			
	Stroma in the invading tumor edge (/×400) (range 0‐200)	0.3	0.3	0.12				0.36	0.12‐1.08	0.06				0.9	0.49‐1.63	0.73			
	Parenchyma in the invading tumor edge (/×400) (range 0‐200)	0.02	0.00‐1.99	0.09				0.22	0.06‐0.75	**0.01**	0.19	0.05‐0.72	**0.01**	0.59	0.32‐1.10	0.10			
	Periphery of the tumor (/×400) (range 0‐200)	0.53	0.15‐1.93	0.34				0.62	0.26‐1.65	0.37				0.36	0.17‐0.75	***P* < 0.01**	0.38	0.18‐0.80	**0.01**
Clinical findings	Gender	1.27	0.44‐3.64	0.64				1.1	0.49‐2.45	0.81				1.4	0.81‐2.43	0.22			
	Age (years)	0.1	0.01‐0.76	0.02				0.18	0.05‐0.60	***P* < 0.01**	0.26	0.07‐0.98	**0.04**	0.47	0.25‐0.87	**0.01**	0.47	0.24‐0.90	**0.02**
	Tumor site	3.8	1.27‐11.37	0.01				3.09	1.37‐6.98	***P* < 0.01**	1.48	0.49‐4.42	0.47	1.6	0.92‐2.80	0.09			
	cT stage	3.39	1.45‐7.91	***P* < 0.01**	0.53	0.25‐2.02	0.53	3.42	1.79‐6.53	***P* < 0.01**	0.83	0.31‐2.21	0.71	1.51	0.97‐2.33	0.06			
	cN stage	7.66	2.56‐22.91	***P* < 0.01**	0.7	0.03‐9.19	0.7	3.69	1.65‐8.26	***P* < 0.01**	0.17	0.03‐0.95	**0.04**	1.59	0.87‐2.91	0.12			
	cTNM stage	4.99	1.94‐12.81	***P* < 0.01**	2.15	0.17‐26.48	0.54	3.41	1.81‐6.42	***P* < 0.01**	3.01	0.70‐12.96	0.13	1.44	0.99‐2.08	0.05			
	Operative method	8.34	2.32‐29.94	***P* < 0.01**	1.84	0.28‐12.08	0.52	3.9	1.70‐8.92	***P* < 0.01**	1.34	0.36‐5.04	0.65	1.19	0.67‐2.10	0.54			
Pathological findings	Histologic grade	2.24	0.95‐5.24	0.06				1.64	0.84‐3.20	0.14				2.08	1.29‐3.34	***P* < 0.01**	1.48	0.92‐2.37	0.1
	pT stage	5.62	2.52‐12.53	***P* < 0.01**	9.56	1.77‐51.67	***P* < 0.01**	3.68	2.09‐6.49	***P* < 0.01**	6.6	1.45‐29.96	**0.01**	1.46	0.98‐2.16	0.05			
	pN stage	12.63	3.95‐40.38	***P* < 0.01**	36.64	2.95‐455.23	***P* < 0.01**	3.76	1.67‐8.50	***P* < 0.01**	6.47	1.40‐29.86	**0.01**	6.32	3.59‐11.11	***P* < 0.01**	6.09	2.36‐15.71	***P* < 0.01**
	pTNM stage	5.76	2.19‐15.16	***P* < 0.01**	0.25	0.02‐2.38	0.23	2.66	1.54‐4.57	***P* < 0.01**	0.3	0.06‐1.38	0.12	2.39	1.65‐3.46	***P* < 0.01**	1.04	0.59‐1.81	0.89
	Lymphovascular invasion	2	0.62‐6.39	0.24				2.1	0.87‐5.08	0.09				2	1.06‐3.75	**0.03**	1.49	0.77‐2.88	0.23
	Perineural invasion	4.44	1.39‐14.20	**0.01**	0.14	0.02‐0.92	**0.04**	3.91	1.54‐9.88	***P* < 0.01**	0.51	0.13‐1.90	0.31	2.72	1.32‐5.62	***P* < 0.01**	0.83	0.35‐1.98	0.68
	Surgical margin status	0.7	0.09‐5.38	0.73				1.89	0.64‐5.55	0.24	0.19			1.43	0.64‐3.18	0.37			

Bold indicates *P* < 0.05.

## 4 DISCUSSION

The key finding from the current study is that previously untreated patients with OSCC with high tumor‐infiltrating CD8^+^ T cells had significantly better DSS, OS, and RFS. This relationship was retained in multivariate Cox regression analysis estimated by including clinicopathological parameters positively associated with OS and RFS. The correlation between TILs and patient survival has been well reported in various types of cancers, including HNSCC.^21^ Of TILs, accumulating evidence shows that CD8^+^ T cells are a key component of antitumor immunity.^22^ High expression of tumor antigens could drive activation of the CD8^+^ T‐cell antitumor response, and depletion of CD8^+^ T cells drives cancer cell growth, underscoring the importance of CD8^+^ T cells in controlling cancer growth.^23^ In the majority of cancer types, CD8^+^ T‐cell infiltrates predict favorable prognosis.^24^, ^25^, ^26^ Meta‐analyses revealed that CD8^+^ T cells have a positive effect on OS, with a HR of 0.71 (95% CI 0.62‐0.82),^27^ and are effective prognostic predictors for OS and DSS in breast cancer.^28^ CD8^+^ T cells were also predictors for OS and disease‐free survival (DFS) in stage I non‐small cell lung cancer.^29^

A recent meta‐analysis on tumor‐infiltrating immune cells suggested that the amount and density of tumor‐infiltrating CD8^+^ T cell also affected survival in HNSCC patients,^30^ whereas there is controversy as to whether higher levels of tumor‐infiltrating CD8^+^ T cells improve survival in patients with OSCC. Several studies indicated that tumor‐infiltrating immune cells did not provide any survival benefit in patients with OSCC.^31^, ^32^ However, these observations were made in a small sample size (under 50 subjects) and a shorter follow‐up duration than used with the present cohort. Those studies also examined different tumor areas.

Some authors have indicated that immune cells infiltration affected OS, DSS, and DFS.^15^, ^19^, ^33^ Higher CD4^+^ cell levels was an independent predictor for improved OS and DSS in 278 patients with HNSCC who received heterogeneous treatment strategies.^18^ In contrast, Balermpas et al,^19^ showed that high CD3^+^ and CD8^+^ T‐cell density were associated with significantly increased OS and PFS in patients receiving definitive chemoradiotherapy, while neither CD4^+^ nor FoxP3^+^ immune cell density showed significance for the clinical outcome. The authors of the present study have previously reported that high stromal T‐cell density increases the effectiveness of neoadjuvant bleomycin therapy in patients with OSCC.^9^ Differences in tumor‐infiltrating T‐cell subsets could influence the effectiveness of cancer treatment. Recently, Tabachnyk et al,^16^ showed that a high density of tumor‐infiltrating CD8^+^ T cells observed in OSCC patients had a better DFS after concurrent chemoradiotherapy followed by surgery. Similar research data with respect to neoadjuvant therapy have been reported in breast cancer.^34^ However, little is known whether adjuvant local and/or systemic cancer therapy could influence the outcomes of studies evaluating CD8^+^ T‐cell infiltration or not. Patients with positive surgical margin in the present study did not receive routine adjuvant therapy.

The present study considered relationships between localization, density of CD8^+^ T‐cell infiltration, clinicopathological parameters, outcome, and prognosis. The relationships between the locations of CD8^+^ T‐cell infiltrates, CD8^+^ T‐cell density, and survival are diverse. Naito et al^13^ observed in patients with colorectal cancer that CD8^+^ T cells located in the tumor stroma or tumor margin did not affect prognosis, whereas only CD8^+^ T cells located in the tumor epithelium affected prognosis positively. On the contrary, Menon et al^35^ showed that marked stromal infiltration of CD8^+^ T cells at the advancing tumor margin was an independent prognostic factor for a longer DFS in colorectal cancer. Bindea et al^36^ reported that higher CD8^+^ T‐cell density was observed in the stroma than in the parenchyma. They also showed that stromal CD8^+^ T‐cell density was higher in the invasive margin than in the center of the tumor, whereas parenchymal and stromal CD8^+^ T‐cell density showed approximately equal densities. Furthermore, their study demonstrated that parenchymal CD8^+^ T‐cell density was significantly associated with a better RFS in colorectal cancer. In OSCC, as shown in the current study, stromal CD8^+^ T‐cell density was higher than parenchymal CD8^+^ T‐cell density in the center of the tumor, as well as in the invading edge.

The specific locations of adaptive cellular immune reaction within tumor samples, especially within the core of the tumor and the invasive margin, are highly significant parameters to predict tumor relapse and survival. Galon et al^25^ investigated the intratumoral adaptive immune response in the center of the tumor and the invasive margin, and they reported originally that the combined analysis of the two tumor regions improved the prediction of colorectal cancer patient survival. Pagès et al^37^ further demonstrated that the combined analysis of CD8^+^ plus CD45RO^+^ cells in the center of the tumor and invasive margin could provide a useful criterion for the prediction of tumor recurrence and survival in patients with early‐stage colorectal cancer. Moreover, Mlecnik et al^38^ concluded that assessing intratumoral CD8^+^ T‐cell density in combined tumor regions, the center of the tumor and the invasive margin, provides an indicator of tumor recurrence beyond that predicted by AJCC/UICC‐TNM staging. At present, TNM staging system is the gold standard for risk assessment for cancers. However, data from the cohort of the present study showed that using parenchymal tumor‐infiltrating CD8^+ ^T‐cell density in the invading tumor edge and peritumoral stroma for predicting cancer recurrence and survival in patients with OSCC was superior to and independent of the cTNM or pTNM staging system. These reactive T cells in specific areas may have a protective effect against cancer proliferation, invasion, and metastasis. These findings in OSCC need to be validated in a larger cohort and with other types of cancer, such as colorectal cancer.

Although the present study focused on tumor‐infiltrating CD8^+ ^T cells in OSCC, the type and functional status of immune cells, including CD4^+^ cells and FoxP3^+^ immunosuppressive T cells, and/or the tissue localizations of different tumor‐infiltrating immune cells can determine the balance between control or promotion of cancer.^12^ One antitumor mechanism employed by cytotoxic T cells requires that the cytotoxic cells physically contact cancer cells.^39^ This type of cytotoxic T cell may invade the tumor parenchyma, especially at the invading tumor edge, as observed in the present study. CD8^+^ T cells can also recognize tumor antigens processed by the stroma,^40^ and activated CD8^+^ T cells secrete cytokines that induce cancer cell senescence and play essential roles in the control of anticancer immune responses and tumor growth.^41^ Thus, it is not surprising that indirect cytotoxic activity of CD8^+^ T cells may occur at peripheral stroma of the tumors.

The tumor microenvironment may regulate the accumulation of T cells in tumors at the initial step of their interaction with local blood vessels.^39^ The presence of tumor‐infiltrating CD8^+^ T cells can predict the response of solid tumors to anti‐PD‐1 monoclonal antibody therapy.^42^ Moreover, clinical and histopathological anticancer effects of chemotherapy and/or concomitant chemoradiotherapy may depend on vascularity in the tumor microenvironment.^43^ Therefore, assessment of CD8^+^ T‐cell density in specific locations in biopsied samples can be used as a novel tool for selecting responders to these treatment strategies. In addition, as described by Bindea et al,^36^ the immune environment in cancer tissues may contain approximately 30 different immune cell types. Therefore, detailed investigation of such immune cells in many types of cancer would be needed.

There are several limitations of our investigation. The retrospective analysis of tumor‐infiltrating CD8^+^ T cells cannot exclude potential selection bias. Moreover, to minimize confounding factors by limiting the assessment of tumor‐infiltrating immune cells to just CD8^+^ T cells in the present study, other immune cell populations in the tumor microenvironment were excluded. Interactions between immune cells, in addition to their clinical significance, needs further investigation.

In summary, the results of this study showed significant associations between increased tumor‐infiltrating CD8^+^ T cells and their tissue localization in OSCC. High stromal CD8^+^ T‐cell density at the periphery of the tumor and high parenchymal CD8^+^ T‐cell density at the invading edge were independent prognostic makers for RFS and OS, respectively. Thus, the present study revealed site‐specific informative features of the CD8^+^ T‐cell infiltration: the parenchyma at invading tumor edge and the peritumor. Pathological immunity evaluation may provide crucial novel prognostic information and help identify patient cohorts likely to benefit from immunotherapy.

## ETHICAL CONSIDERATIONS

All procedures complied with the ethical standards of the relevant local and national committees on human experimentation and with the latest version of the Helsinki Declaration of 1964. Informed consent or acceptable substitute was obtained from all patients before study inclusion.

## ETHICAL APPROVAL AND CONSENT TO PARTICIPATE

This retrospective study was conducted according to the principles stated in the 1964 Declaration of Helsinki and its subsequent versions and was approved by the Institutional Review Board of our university on 12 September 2017 (No. 292‐1116). Informed consent or acceptable substitute was obtained all patients before study inclusion.

## CONFLICT OF INTEREST

None declared.
